# Radiomics versus Visual and Histogram-based Assessment to Identify Atheromatous Lesions at Coronary CT Angiography: An ex Vivo Study

**DOI:** 10.1148/radiol.2019190407

**Published:** 2019-08-06

**Authors:** Márton Kolossváry, Júlia Karády, Yasuka Kikuchi, Alexander Ivanov, Christopher L. Schlett, Michael T. Lu, Borek Foldyna, Béla Merkely, Hugo J. Aerts, Udo Hoffmann, Pál Maurovich-Horvat

**Affiliations:** From the MTA-SE Cardiovascular Imaging Research Group, Heart and Vascular Center, Semmelweis University, 68 Varosmajor St, 1122 Budapest, Hungary (M.K., J.K., B.M., P.M.H.); Department of Radiology, Massachusetts General Hospital, Harvard Medical School, Boston, Mass (J.K., Y.K., A.I., M.T.L., B.F., H.J.A., U.H.); Center for Cause of Death Investigation, Faculty of Medicine, Hokkaido University, Hokkaido, Japan (Y.K.); Department for Diagnostic and Interventional Radiology, Medical Center-University of Freiburg, Freiburg, Germany (C.L.S.); and Department of Radiation Oncology, Dana-Farber Cancer Institute, Brigham and Women’s Hospital, Harvard Medical School, Boston, Mass (H.J.A.).

## Abstract

**Background:**

Visual and histogram-based assessments of coronary CT angiography have limited accuracy in the identification of advanced lesions. Radiomics-based machine learning (ML) could provide a more accurate tool.

**Purpose:**

To compare the diagnostic performance of radiomics-based ML with that of visual and histogram-based assessment of ex vivo coronary CT angiography cross sections to identify advanced atherosclerotic lesions defined with histologic examination.

**Materials and Methods:**

In this prospective study, 21 coronary arteries from seven hearts obtained from male donors (mean age, 52.3 years ± 5.3) were imaged ex vivo with coronary CT angiography between February 23, 2009, and July 31, 2010. From 95 coronary plaques, 611 histologic cross sections were coregistered with coronary CT cross sections. Lesions were considered advanced if early fibroatheroma, late fibroatheroma, or thin-cap atheroma was present. CT cross sections were classified as showing homogeneous, heterogeneous, or napkin-ring sign plaques on the basis of visual assessment. The area of low attenuation (<30 HU) and the average Hounsfield unit were quantified. Radiomic parameters were extracted and used as inputs to ML algorithms. Eight radiomics-based ML models were trained on randomly selected cross sections (training set, 75% of the cross sections) to identify advanced lesions. Visual assessment, histogram-based assessment, and the best ML model were compared on the remaining 25% of the data (validation set) by using the area under the receiver operating characteristic curve (AUC) to identify advanced lesions.

**Results:**

After excluding sections with no visible plaque (*n* = 134) and with heavy calcium (*n* = 32), 445 cross sections were analyzed. Of those 445 cross sections, 134 (30.1%) were advanced lesions. Visual assessment of the 445 cross sections indicated that 207 (46.5%) were homogeneous, 200 (44.9%) were heterogeneous, and 38 (8.5%) demonstrated the napkin-ring sign. A radiomics-based ML model incorporating 13 parameters outperformed visual assessment (AUC = 0.73 with 95% confidence interval [CI] of 0.63, 0.84 vs 0.65 with 95% CI of 0.56, 0.73, respectively; *P* = .04), area of low attenuation (AUC = 0.55 with 95% CI of 0.42, 0.68; *P* = .01), and average Hounsfield unit (AUC = 0.53 with 95% CI of 0.42, 0.65; *P* = .004) in the identification of advanced atheromatous lesions.

**Conclusion:**

Radiomics-based machine learning analysis improves the discriminatory power of coronary CT angiography in the identification of advanced atherosclerotic lesions.

Published under a CC BY 4.0 license.

## Graphical abstract

**Figure:**
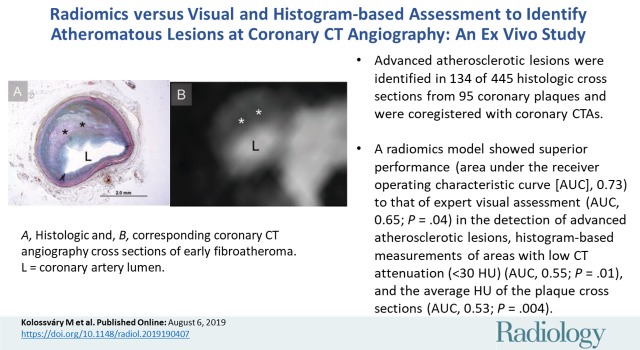

**Summary**

Radiomics-based machine learning of coronary CT angiography in the identification of advanced atherosclerotic lesions has higher diagnostic accuracy compared with visual and histogram-based assessment.

**Key Results**

■ Advanced atherosclerotic lesions (ie, the presence of early fibroatheroma, late fibroatheroma, or thin-cap atheroma) were identified in 134 of 445 histologic cross sections from 95 coronary plaques and were coregistered with coronary CT angiograms.
■ Expert visual assessment of the CT angiograms showed moderate diagnostic performance in the detection of advanced atherosclerotic lesions (area under the receiver operating characteristic curve [AUC] = 0.65).
■ A radiomics-based machine learning model showed superior performance (AUC = 0.73) to that of expert visual assessment (AUC = 0.65, *P* = .04), histogram-based measurements of areas with low CT attenuation (<30 HU) (AUC = 0.55, *P* = .01), and the average Hounsfield units of the plaque cross sections (AUC = 0.53, *P* = .004).

## Introduction

The modified American Heart Association classification scheme categorizes coronary atherosclerotic plaques into six histologic classes: adaptive intimal thickening, pathologic intimal thickening, fibrous plaque, early fibroatheroma, late fibroatheroma, and thin-cap fibroatheroma (1,2). Adaptive and pathologic intimal thickening and fibrous plaque are considered early atherosclerotic lesions, whereas early and late fibroatheromas and thin-cap fibroatheroma are advanced atherosclerotic lesions associated with a higher risk of myocardial infarction (3–7). Therefore, the ability to noninvasively identify advanced lesions could improve risk stratification and prediction of future cardiovascular events.

Coronary CT angiography can help characterize the morphologic characteristics and composition of plaques noninvasively. Noncalcified and partially calcified plaques can be differentiated into homogeneous, heterogeneous, and napkin-ring sign plaques on the basis of visual assessment of the CT attenuation pattern of the noncalcified plaque region (8,9). The plaque attenuation pattern scheme outperforms conventional plaque classification (calcified, partially calcified, and noncalcified) to identify advanced atherosclerotic lesions with use of histologic findings as the reference standard (10). Recently, quantitative histogram analysis and the area or volume of a low-attenuation plaque have been proposed as markers of high-risk lesions (11–13). However, radiologic images contain much more information than what we can comprehend visually or quantify with simple manual measurements (14–16).

Radiomics represents a process of extracting thousands of imaging markers from radiologic images describing the heterogeneity and spatial complexity of lesions (17). These quantitative features can be used as the input to machine learning (ML). We hypothesized that ML analysis of radiomic parameters would outperform visual assessment and histogram-based methods in the identification of advanced atherosclerotic lesions. Therefore, our objective was to compare the diagnostic performance of radiomics-based ML with that of visual and histogram-based plaque assessment in the identification of advanced coronary lesions with use of histologic examination as a reference standard.

## Materials and Methods

### Study Design

In this prospective study, images were acquired between February 23, 2009, and July 31, 2010. The same data set has been previously used to investigate the effects of image reconstruction and delineation method on coronary plaque assessment (18–20); to compare coronary CT angiography, intravascular US, and optical coherence tomography imaging markers of advanced atherosclerotic lesions (21); and to assess the value and histologic predictors of the napkin-ring sign (10,22). In this study, we wished to assess whether radiomics-based ML could outperform the previously described napkin-ring sign. Our study was approved by the institutional ethics committees and was performed in accordance with the Health Insurance Portability and Accountability Act, local and federal regulations, and the Declaration of Helsinki. Written informed consent was acquired from the donor or next of kin. The authors had full control of the data and the information submitted for publication.

### Study Cohort

Donor hearts were provided by the International Institute for the Advancement of Medicine (Jessup, Penn). Donor hearts were included if they were from men between the ages of 40 and 70 years with a history of myocardial infarction or coronary artery disease proven with diagnostic tests (10,21). Donors with coronary artery bypass grafts were excluded. The maximum allowed warm and cold ischemia time was 6 and 15 hours, respectively. Overall, seven ex vivo donor hearts (mean donor age ± standard deviation: 52.3 years ± 5.3) were investigated. The cause of death was stroke (*n* = 6) and suicide (*n* = 1).

### Heart Preparation and Coronary CT Angiography

The heart preparation was described previously (10,21). The fresh hearts were imaged without formalin fixation by using a 64–detector row CT scanner (High-Definition, GE Discovery, or CT 750HD; GE Healthcare, Milwaukee, Wis). For coronary CT angiography, a 3% mixture of iodinated contrast material (iopamidol [Isovue 370; Bracco Diagnostics, Milan, Italy]) and methylcellulose (Methocel; Dow Chemical, Midland, Mich) was used. All data sets were acquired in the sequential acquisition mode with collimation of 64 × 0.625 mm, rotation time of 0.35 second, tube voltage of 120 kV, and tube current–time product of 500 mAs. The images were reconstructed with a section thickness of 0.6 mm and an increment of 0.4 mm by using an adaptive iterative reconstruction technique (ASIR, GE Healthcare). Images from coronary CT angiography were analyzed with an offline workstation (Leonardo; Siemens Healthcare, Erlangen, Germany). After CT imaging, the coronary arteries were excised with surrounding tissue and the side branches were ligated. Specimen preparation and coronary CT angiography were performed within 4 hours after receiving the heart to avoid potential postmortem tissue changes.

### Histologic Classification

**Figure 1:**
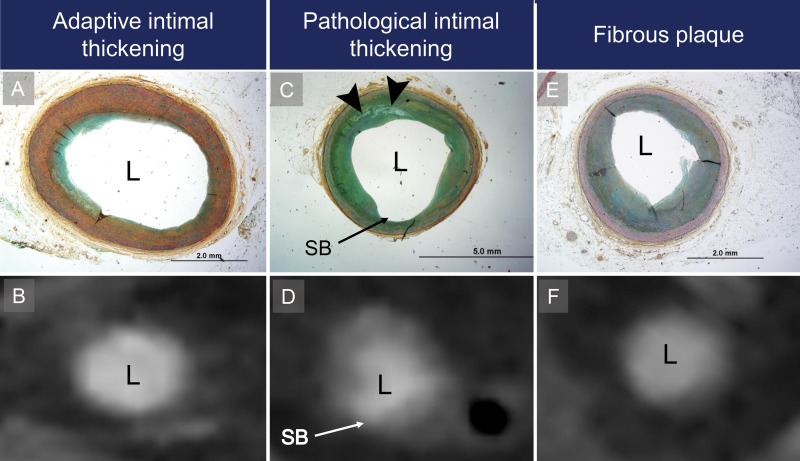
Representative histologic and coronary CT angiography cross sections of early atherosclerotic plaques demonstrate the three types of early atherosclerotic lesions. L = coronary artery lumen. *A*, Histologic and, *B*, coronary CT angiography cross sections of adaptive intimal thickening. *C*, Histologic cross section of pathologic intimal thickening with focal lipid accumulation in arterial wall (arrowheads) and, *D*, corresponding coronary CT angiography cross section. SB = coronary artery side branch. *E*, Histologic and, *F*, corresponding coronary CT angiography cross sections of fibrous plaque.

**Figure 2:**
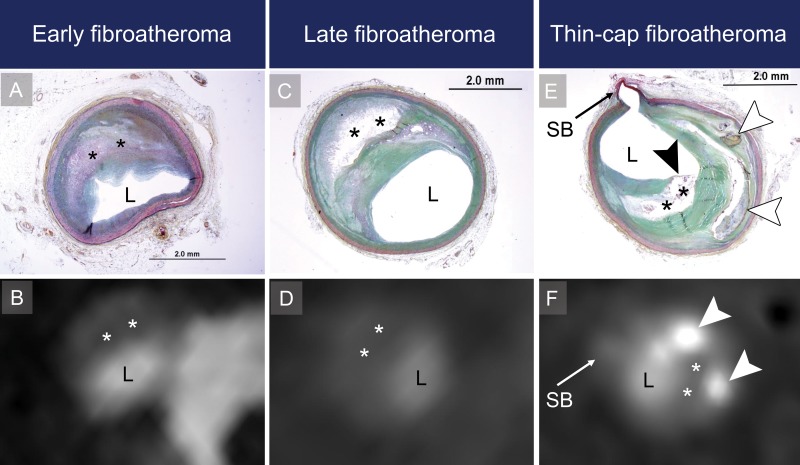
Representative histologic and coronary CT angiography cross sections of advanced atherosclerotic lesions demonstrate the three types of advanced atherosclerotic lesions. L = coronary artery lumen, * = necrotic core. *A*, Histologic and, *B*, corresponding coronary CT angiography cross sections of early fibroatheroma. *C*, Histologic and, *D*, corresponding coronary CT angiography cross sections of late fibroatheroma. *E*, Histologic and, *F*, corresponding coronary CT angiography cross sections of thin-cap fibroatheroma. Black arrowhead indicates the shoulder of the thin-cap, and white arrowheads indicate calcification. SB = coronary artery side branch.

Histologic preparation and analysis were performed at a pathology institute that specializes in cardiovascular histopathology by a certified pathologist with more than 20 years of experience (CVPath Institute, Gaithersburg, Md). Coronary arteries were embedded in paraffin and sliced in 1.5- and 2-mm increments (382 and 185 cuts, respectively). Coronary artery segments with no visible atherosclerotic disease were sliced every 5 mm (44 cuts). The thickness of a single histologic slice was 6 μm. All slices were stained with Movat pentachrome (23). Each cross section was classified according the modified American Heart Association scheme into the following categories: adaptive intimal thickening, pathologic intimal thickening, fibrous plaque, early fibroatheroma, late fibroatheroma, and thin-cap fibroatheroma (Figs 1, 2) (1,2). Adaptive and pathologic intimal thickening and fibrous plaque were considered early atherosclerotic lesions, and early and late fibroatheroma and thin-cap fibroatheroma were categorized as advanced lesions.

### Visual Coronary CT Angiography Analysis

The method of coregistration of histologic slides with coronary CT angiography images was described previously (10). An experienced reader (P.M.H., with 6 years of experience in coronary CT angiography) performed the qualitative reading of all coronary CT angiography cross sections (training and validation data set) and classified them on the basis of the traditional and the plaque attenuation pattern scheme as described in detail previously (10). All readings were done blinded to the histologic results and were performed with a fixed window setting (window width, 700 HU; level, 200 HU).

### Histogram-based Coronary CT Angiography Analysis

All coronary CT angiography cross sections were manually segmented (J.K., with 5 years of experience in coronary CT angiography) by using a dedicated open-source software (3D Slicer, version 4.8.1; *https://www.slicer.org/*) according to the direct plaque tracing method (20,24). Plaque segmentations were used as a mask image to select the voxels that contained plaque. Images were exported in NRRD files and imported into the open-source Radiomics Image Analysis software package (version 1.4.1; *https://CRAN.R-project.org/package=RIA*) in the R environment (25). For histogram-based assessment, the area of low attenuation (<30 HU) and the average Hounsfield units were calculated from the segmented coronary CT angiography images.

### Radiomic Analysis

Segmented image voxel values were then discretized into 16, 32, and 64 equally sized (having identical Hounsfield unit ranges) and equally probable (having the same amount of voxels) bins by using the Radiomics Image Analysis software, resulting in six additional images (25,26). Overall, 44 first-order statistics (describing the Hounsfield unit distribution of the lesion) were calculated on the original voxel values. There were 114 gray-level co-occurrence matrix parameters (describing how often voxels with similar value co-occur next to each other) and 11 gray-level run length matrix parameters (describing how many similar-value voxels are next to each other) on the discretized images, resulting in 6 × 114 = 684 and 6 × 11 = 66 parameters. Among geometry-based parameters (describing the spatial characteristics of the lesions, eg, smoothness or self-symmetry), surface, surface ratio of a specific discretized value to total surface and fractal box counting, and information and correlation dimensions were calculated on the original image and each value of the discretized image, resulting in 5 + (2 × 16 × 5) + (2 × 32 × 5) + (2 × 64 × 5) = 1125 parameters. Altogether, 1919 parameters were calculated for each cross section. A detailed description of each radiomic parameter has been published previously (15,17).

### ML Model Building

For unbiased estimates of diagnostic accuracy, our data set was randomly split into a training set and a validation set. Eight independent ML models were trained on the training set. To select the best model and the best hyperparameters for each model, we conducted five-fold cross-validation on the training set. This technique randomly selects 80% of the data set on which the models are trained and then evaluates them on the remaining 20% (tuning set). This is repeated five times until each cross section was part of the tuning set once. Diagnostic performance was assessed by using the area under the receiver operating characteristic curve (AUC). The average of the five AUC values during cross-validation was used to describe the discriminatory power of that specific model using the given hyperparameter values. This process was repeated 1000 times for each type of ML model by assigning random values to the hyperparameter values by using randomized grid search (27,28).

Model building consisted of the following steps: *(a)* exclusion of parameters with 0 variance, *(b)* robust scaling of parameters by using the median and interquartile range, *(c)* selecting parameters that were under a random significance level (hyperparameter) by using the false-positive rate test or the family-wise error rate test, *(d)* conducting principal component analysis describing a randomly assigned (hyperparameter) portion of the variance, and *(e)* fitting the given ML model with randomly assigned hyperparameters. The following types of ML models were fitted: logistic regression, K-nearest neighbors, random forest, least angle regression, naive Bayes, Gaussian process classifier, decision trees, and deep neural networks (27,28). The ML model that provided the best results on the training set was then applied to the validation set. In case of the plaque attenuation pattern scheme and histogram-based measurements, a logistic regression model was fitted to the training set, which was then applied to the validation set to evaluate discriminatory power. All model building was done in the Python environment (version 3.6.2, Beaverton, Ore; *https://www.python.org/*) by using the Scikit-learn package (version 0.19.1; *https://scikit-learn.org/*) (27). Code used for analysis can be accessed at *https://github.com/martonkolossvary/radiomics_ex-vivo_src**.*

### Statistical Analysis

Continuous variables are presented as averages and standard deviations, and categorical variables are reported as frequencies and percentages. Categorical variables were compared by using the χ^2^ test. Diagnostic accuracy of visual assessment, histogram-based methods, and radiomics-based ML were evaluated on the validation set by using receiver operating characteristic curves. The 95% confidence intervals (CIs) of the AUCs were calculated, and the receiver operating characteristic curves were compared by using the DeLong method (29).

All statistical calculations were done in Python and R (version 3.4.2, Vienna, Austria; *https://www.r-project.org/*) environments. Two-sided *P* < .05 was considered indicative of a statistically significant difference.

## Results

The demographic characteristics of the heart donors are summarized in Table 1.

**Table 1:**
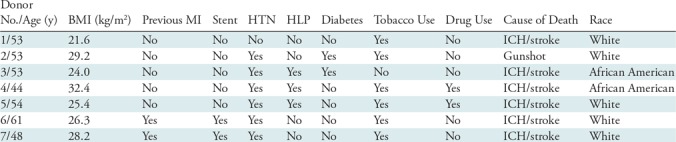
Donor Characteristics

Note.—All donors were men. BMI = body mass index, HLP = hyperlipidemia, HTN = hypertension, ICH = intracerebral hemorrhage, MI = myocardial infarction.

### Distribution of Histologic and CT Categories of Cross Sections

Overall, we evaluated 611 histologic slices from 21 coronary arteries of seven donor hearts. The average vessel length was 67 mm (range, 25–110 mm). Of the 611 sections, 71 (11.6%) were identified as adaptive intimal thickening, 222 (36.3%) as pathologic intimal thickening, 179 (29.3%) as fibrous plaque, 59 (9.7%) as early fibroatheroma, 60 (9.8%) as late fibroatheroma, and 20 (3.3%) as thin-cap fibroatheroma; 477 (78.1%) contained plaque that was detectable at CT angiography. Noncalcified plaque was present in 254 of those 477 cross sections (53.2%), partially calcified plaque was present in 191 (40.0%), and calcified plaque was present in 32 (6.7%). Because plaque attenuation pattern–based classification is based on noncalcified plaque components, all analyses were performed by excluding cross sections containing purely calcified lesions. Therefore, 445 cross sections were analyzed.

The data set was randomly split into a training set (333 of 445 cross sections, 75%) and a validation set (112 of 445 cross sections, 25%). The distribution of histologic and CT angiography categories of the analyzed plaques can be found in Table 2. There was no difference between the training set and validation set with regard to the distribution of early or advanced atherosclerotic lesion categories and plaque types according to either a traditional CT angiography classification scheme or a plaque attenuation classification scheme (early atherosclerotic lesions: *P* = .90; advanced atherosclerotic lesions: *P* = .71; traditional plaque classification: *P* = .26; plaque attenuation pattern: *P* = .41).

**Table 2:**
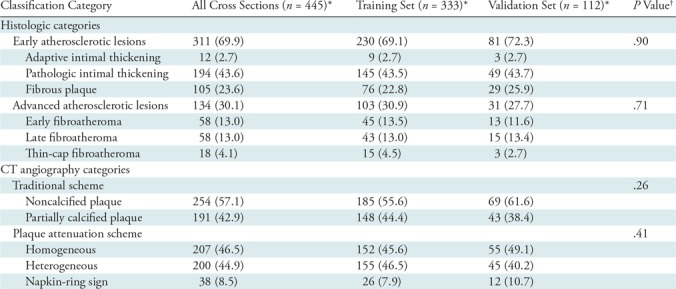
Distribution of Histologic and CT Angiography Categories of Analyzed Cross Sections

*Data are numbers of cross sections, with percentages in parentheses.

^†^*P* values correspond to comparisons between the training and validation sets.

The distribution of early and advanced atherosclerotic lesions in noncalcified and partially calcified plaques was similar for all cross sections (*P* = .08). Conversely, the distribution of CT angiography cross sections showing homogeneous, heterogeneous, and napkin-ring sign attenuation patterns differed between early and advanced atherosclerotic lesions (*P* < .001). Detailed results can be found in Table 3.

**Table 3:**
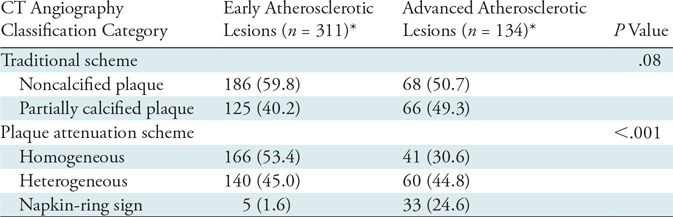
Frequency of Traditional and Plaque Attenuation–based CT Angiography Categories for Early and Advanced Atherosclerotic Lesions

*Data are numbers of cross sections, with percentages in parentheses.

### Identification of Advanced Atherosclerotic Lesions with Coronary CT Angiography

**Figure 3:**
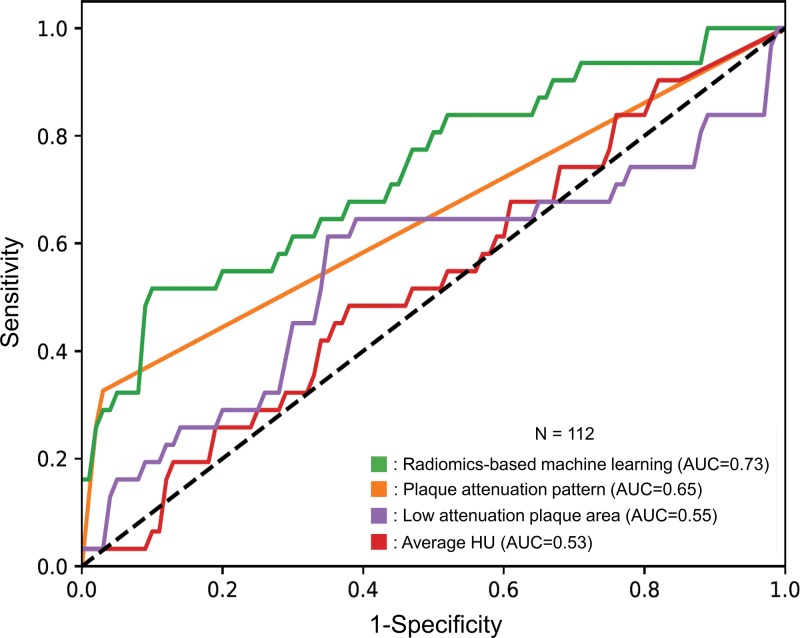
Receiver operating characteristic curves of radiomics-based machine learning (ML) model, plaque attenuation pattern, area of low attenuation, and average Hounsfield unit in the identification of advanced atherosclerotic lesions. Radiomics-based ML method showed the best discriminatory power (area under the receiver operating characteristic curve [AUC] = 0.73; 95% confidence interval [CI]: 0.63, 0.84). The discriminatory power of visual assessment with use of the plaque attenuation pattern was worse than that of the ML model (AUC = 0.65 [95% CI: 0.56, 0.73]; *P* = .04). The histogram-based methods area of low attenuation and average Hounsfield unit showed poor diagnostic accuracy compared with the radiomics-based ML model (AUC = 0.55 [95% CI: 0.42, 0.68] for area of low attenuation and 0.53 [95% CI: 0.42, 0.65] for average Hounsfield unit; *P* = .01 and *P* = .004).

Among radiomics-based ML models, the least angles regression model provided the best discriminatory power on the training set. Diagnostic accuracies of the radiomics-based ML models on the training set can be found in Table 4. The following hyperparameters for the processing pipeline produced the best results on the training set using the least angles regression model. First, we excluded all zero variance parameters and scaled the parameters based on medians and interquartile ranges. Next, the best predictors based on the training set were selected by using significance levels of α = .05 and α = .0007 for the family-wise error rate and false-positive rate test, respectively. Then, we conducted principal component analysis to construct derived parameters explaining the 95% of the variation in the data. Afterward, 13 parameters were selected to be inputs to the least angle regression model. This fitted model was then applied to the validation set to evaluate the unbiased discriminatory power of the model. The radiomics-based ML model achieved good diagnostic accuracy (AUC = 0.73 [95% CI: 0.63, 0.84]) on the validation set. The plaque attenuation pattern scheme achieved moderate diagnostic accuracy (AUC = 0.65 [95% CI: 0.56, 0.73]), whereas the histogram-based measurements area of low attenuation (<30 HU) and average Hounsfield units of the plaque cross sections produced poor diagnostic accuracy (AUC = 0.55 [95% CI: 0.42, 0.68] and 0.53 [95% CI: 0.42, 0.65], respectively) on the validation set. The radiomics-based ML model outperformed expert visual assessment (AUC = 0.73 vs 0.65; *P* = .04) and the histogram-based measurements area of low attenuation (<30 HU) (AUC = 0.73 vs 0.55, *P* = .01) and average Hounsfield units of the plaque cross sections (AUC = 0.73 vs 0.53, *P* = .004). Receiver operating characteristic curves of the corresponding models evaluated on the validation set are shown in Figure 3.

**Table 4:**
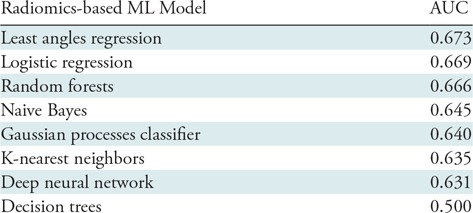
Discriminatory Power of Radiomics-based ML Models on the Training Set in the Identification of Advanced Atherosclerotic Lesions

Note.—The least angles regression model provided the best discriminatory power; therefore, the fitted model was applied to the validation set to evaluate true diagnostic accuracy. ML = machine learning.

## Discussion

From seven ex vivo hearts, we trained a radiomics-based machine learning model on a training set (333 of 445 cross sections, 75%) of coronary CT angiography images to identify advanced atherosclerotic lesions. When we evaluated our results on a separate validation set (112 of 445 cross sections, 25%), we found that radiomics-based machine learning can better differentiate between early and advanced atherosclerotic lesions as compared with the plaque attenuation pattern scheme in CT angiography cross sections (area under the receiver operating characteristic curve [AUC]: 0.73 vs 0.65, respectively; *P* = .04) and the histogram-based measurements area of low attenuation (<30 HU) (AUC = 0.73 vs 0.55; *P* = .01) and average Hounsfield units of the plaque cross sections (AUC = 0.73 vs 0.53; *P* = .004).

Large necrotic cores of advanced atherosclerotic lesions carry an inherently higher risk of plaque rupture; therefore, the identification of these lesions is of utmost importance (7). Coronary CT angiography would be an ideal imaging modality to identify these advanced lesions owing to its noninvasive nature and wide-spread availability. It has previously been shown that the plaque attenuation pattern–based scheme outperforms conventional classification in the identification of advanced atherosclerotic lesions (AUC: 0.76 vs 0.68, respectively; *P* = .001) (10). However, the previous investigation assessed the diagnostic accuracy on the whole data set; therefore, those results were overly optimistic and preclude the generalization of the results to other populations. In addition, because the reproducibility of qualitative imaging markers is poor even among experienced readers, the generalizability of results based on visual assessment is limited (30). More objective methods with less reliance on reader experience are warranted.

To overcome the limitations of visual assessment and to provide a more objective method to characterize atherosclerotic plaques, quantitative histogram-based methods based on Hounsfield unit measurements have been proposed (11–13). Previous results show a good correlation between quantified low-attenuation plaque volume or area and the presence of large lipid cores (11,18,31). However, it is not only the presence of lipid-rich plaque components that defines advanced atherosclerotic lesions but also the spatial distribution of various tissue components (10). In addition, these methods are limited by the fact that different tissue components may have overlapping Hounsfield units (31).

Radiomics has been shown to identify napkin-ring sign plaques with excellent diagnostic accuracy (15). Similarly, MRI may also be used to classify atherosclerotic lesions, and radiomic analysis of images from MRI has been shown to differentiate between acute or subacute symptomatic and asymptomatic plaques in the basilar artery (32–34). Furthermore, ML has proven to be a valuable tool in medical data analysis (35,36), identifying insights from big data databases by using alternative statistical techniques. Instead of using conventional statistical methods, these procedures are based on methods originating from how we learn and perceive our surroundings (37). It seems that ML is helpful in medical image analysis too, as our results indicate that applying ML to radiomic features from coronary CT angiography images outperforms current methods. Although our AUCs may appear to some as limited, we want to emphasize that based on recent data demonstrating that local plaque composition has low positive predictive value in the identification of locations of future plaque rupture leading to myocardial infarction (38), our radiomics marker may be used as an additional tool to refine risk stratification and tailor medical therapy. For this purpose, our AUCs appear to be acceptable and useful.

Our study has limitations. Our results are based on coronary CT angiography images acquired from a motion-free ideal environment; therefore, the translation of our results to in vivo environments might be limited. Furthermore, despite the relatively large number of cross sections, our analysis is based on only seven hearts. In addition, the training and validation sets consisted of cross sections from the same individuals, which might have biased our results. However, we chose to randomly select our validation data set on a per–cross section basis rather than at an individual level because selecting only one or two cases for validation might not represent the general population well. Furthermore, to overcome overfitting of our models, we evaluated diagnostic performance on a separate validation set. Moreover, the overall number of advanced atherosclerotic lesions and especially thin-cap fibroatheromas in our data set was small; however, this is representative of general populations. In addition, we did not analyze purely calcified plaques because the partial volume effect of the calcium prohibits analysis of soft-tissue components and, therefore, our results are not generalizable to all plaque types. Furthermore, our radiomics results are based on images from one scanner, reconstruction, and filter setting; therefore, generalizability of the results beyond these settings is unknown. Finally, manual segmentation was a prerequisite for the generation of regions of interest that served as the inputs for histogram and radiomics models.

In conclusion, our results show that radiomics-based machine learning outperformed expert visual assessment and histogram-based methods in the identification of advanced atherosclerotic lesions. Despite the limited spatial resolution of coronary CT angiography, the implementation of machine learning to radiomic features can improve the diagnostic accuracy of coronary CT angiography in the identification of high-risk atherosclerotic lesions and therefore could help in the risk stratification of patients.
